# A non-antibiotic antimicrobial drug, a biological bacteriostatic agent, is useful for treating aerobic vaginitis, bacterial vaginosis, and vulvovaginal candidiasis

**DOI:** 10.3389/fmicb.2024.1341878

**Published:** 2024-05-27

**Authors:** Zhen Zeng, Pei Li, Jiayi Lu, Xiaoqi Li, Meng Li, Yifan Wu, Minzi Zheng, Yang Cao, Qinping Liao, Zhaojia Ge, Lei Zhang

**Affiliations:** ^1^Department of Obstetrics and Gynecology, School of Clinical Medicine, Beijing Tsinghua Changgung Hospital, Tsinghua University, Beijing, China; ^2^College of Life Science, Qingdao Agricultural University, Qingdao, China; ^3^Institute for Precision Medicine, Tsinghua University, Beijing, China

**Keywords:** biological bacteriostatic agent, vaginal infections, non-antibiotic, vaginitis, antimicrobial drug

## Abstract

**Background:**

Vaginitis is a common infection in women, with approximately 75% of women experiencing at least one episode during their lifetime. Although antimicrobial agents are widely used to treat vaginitis, recurrent vaginitis occurs in some patients. Resistance to these agents is the major cause of recurrent vaginitis. Therefore, there is an urgent need to develop novel drugs.

**Methods:**

We investigated the efficacy of a new biological bacteriostatic agent (BBA), composed of lysozyme, phytoalexin, chitosan oligosaccharide, sinensetin, 18β/20α-glycyrrhizin, and betaine, against vaginitis using *in vitro* and *in vivo* studies. First, we evaluated the antibacterial effects of BBA against 13 microbial strains commonly present in aerobic vaginitis, bacterial vaginosis, vulvovaginal candidiasis, and healthy vaginas. Second, we assessed the safety of various doses of BBA administered orally for 4 weeks in female mice. Third, we examined the *in vivo* anti-proliferative and anti-inflammatory effects of BBA in *Candida albicans-*, *Candida glabrata*-, and *Gardnerella*-induced vaginitis models. Finally, we evaluated the anti-vaginitis effect of a BBA gel prepared with 0.5% (w/v) ammonium acryloyldimethyltaurate/Vp copolymer.

**Results:**

BBA effectively suppressed the growth of the main causative pathogens of vaginitis *in vitro*. BBA, either undiluted or diluted two-fold, inhibited all microorganisms cultured for 8 h. No obvious organ damage was detected when BBA was administered to mice. Both BBA alone and 70% BBA in a gel formulation effectively inhibited the proliferation of *C. albicans*, *C. glabrata*, and *Gardnerella* in vaginal lavage samples and alleviated tissue inflammation in mice with vaginitis. The 70% BBA gel performed better than BBA alone at treating vaginitis in mice infected with *Gardnerella vaginalis*.

**Conclusion:**

BBA alone and a 70% BBA gel inhibited the growth of pathogens and effectively alleviated inflammation caused by *C. albicans*, *C. glabrata*, and *G. vaginalis*.

## Highlights

- Our study demonstrated that a new biological bacteriostatic agent (BBA) developed from natural products was safe and effective at suppressing the growth of most pathogens *in vitro* and alleviating *C. albicans-*, *C. glabrata-*, and *Gardnerella*-induced vaginitis *in vivo*.
- Considering its potential for future clinical translation, BBA as a gel formulation is recommended. The 70% BBA gel showed the greatest antimicrobial effect *in vitro* and a better therapeutic effect than BBA against *Gardnerella vaginalis*-induced bacterial vaginosis *in vivo*.

## 1 Introduction

Vaginitis is a common gynecological infection that can lead to anxiety, shame, concerns about hygiene, and decreased quality of life. Most women experience at least one episode of vaginitis during their lifespan (Yadav et al., 2019). The most common cause of vaginitis is vaginal dysbiosis, which increases the risk of bacterial vaginosis (BV), vulvovaginal candidiasis (VVC), aerobic vaginitis (AV), and trichomoniasis infections (Mitra et al., 2016; van de Wijgert, 2017). Currently, metronidazole, clindamycin, and azoles are commonly used for the treatment of vaginitis, with satisfactory initial cure rates (Paladine and Desai, 2018). However, the unsatisfying treatment effect and extremely high recurrence rate are concerns for both clinicians and patients. In the clinic, approximately 50.0–75.5% of women with BV experience recurrence during their lifetime (Lev-Sagie et al., 2019; Muñoz-Barreno et al., 2021). Previous studies have suggested that reinfection through sexual transmission, biofilm formation, altered *lactobacilli* loads, and antimicrobial resistance are the main causes of recurrent vaginitis (Machado and Cerca, 2015; Bradshaw and Sobel, 2016; Pacha-Herrera et al., 2020; Atiencia-Carrera et al., 2022). Therefore, it is of the utmost importance to explore novel strategies for the treatment of vaginitis.

A healthy vaginal environment is maintained by balancing the vaginal microbiome, in which *Lactobacillus* is the dominant bacterial component. The metabolites of lactobacilli, such as lactic acid, bacteriocins, and hydrogen peroxide, maintain the vaginal pH value at 3.8–4.4, which is unfavorable for facultatively pathogenic microorganisms (Chee et al., 2020). When the vaginal microecological environment is disrupted, protection of the vaginal epithelium is reduced, and pathogenic/opportunistic microorganisms gradually replace certain *Lactobacillus* species, leading to infection (Machado et al., 2022). BV is characterized by alterations in the vaginal environment and a shift in the vaginal microbiota from *Lactobacillus* species to a high bacterial diversity, including facultative anaerobes. *Gardnerella* are the characteristic bacteria in the vagina of patients with BV and the key bacteria in the pathogenesis of BV (Salinas et al., 2020). AV, with a reported incidence ranging from 2.0 to 25.8% (Donders et al., 2009), is often associated with other types of vaginitis (Fan et al., 2013; Sonthalia et al., 2020), and the pathogenic microorganisms are mainly *Escherichia coli, Enterococcus* spp., *Streptococcus angina,* and *Streptococcus agalactiae*. However, BV and AV have similar causes, namely, the lack of *Lactobacillus* species (Pacha-Herrera et al., 2020; Zeng et al., 2023). Therefore, probiotics containing *Lactobacillus* strains are a potential option for the treatment of vaginitis. A study conducted by Pacha-Herrera et al. showed a strong multi-microbial consortium comprising *L. iners, L. jensenii, L. gasseri,* and *L. acidophilus* was effective against AV and BV, but was not effective in the absence of *L. gasseri* and *L. acidophilus* (Pacha-Herrera et al., 2022). Probiotics also prevent VVC infections (Lev-Sagie et al., 2019; Tidbury et al., 2021). However, although probiotics are commonly combined with antibiotics to treat vaginitis, they do not completely prevent recurrence (Muñoz-Barreno et al., 2021; Rodríguez-Arias et al., 2022).

Natural product extracts from medicinal plants show potential in preventing the recurrence of vaginitis. Medicinal plants have been used to treat a wide range of infectious diseases for a long time (Azimi et al., 2011; Rashidipour et al., 2022). The antibacterial, anti-inflammatory, antioxidant, and antifungal activities of these herbs have been demonstrated in previous studies. Medicinal plants, such as *Anacyclus pyrethrum, Cymbopogon* species, *Iris germanica, Marrubium vulgare, Myrtus communis, Piper species, Punica granatum,* and *Quercus infectoria* have been used to treat vaginitis (Khalilzadeh et al., 2019; Zhao et al., 2021). Berberine, an herbal alkaloid, inhibits the adhesion of *C. albicans* to vaginal epithelial cells (Ma et al., 2019; Zhao et al., 2022). Polypeptide-enriched *Gastrodia elata* extracts effectively inhibit the proliferation of *C. albicans* and alleviate inflammation (Cai et al., 2019). All these studies suggest that the use of natural product extracts from medicinal plants may be an effective strategy for vaginitis.

In the present study, we developed a natural formulation, a biological bacteriostatic agent (BBA), using natural products extracted from plants, and investigated its efficacy for vaginitis. The main natural components of BBA are lysozyme, phytoalexin, chitosan oligosaccharide, sinensetin, 18β/20α-glycyrrhizin, and betaine. These natural components were extracted from wheat, barley, citrus, licorice, egg whites, and shrimp/crab shells.

## 2 Methods

### 2.1 *In vitro* study

#### 2.1.1 Strain culture

All 13 microbial strains used in this study were obtained from the American Type Culture Collection (ATCC). *Lactobacillus crispatus* TL1J0136, *Lactobacillus jensenii* TL1J0062, and *Lactobacillus gasseri* TL1J0081 were cultured at 37°C in DeMan, Rogosa, and Sharpe medium (BD Difco, Franklin Lakes, NJ, United States; 288210); *Escherichia coli* ATCC 25922, *Staphylococcus aureus* ATCC 25923, and *Enterococcus faecalis* TL2I0352 were cultured at 37°C on tryptone soy agar plates (BD Difco, 211825); *Lactobacillus iners* TL1J0546, *Streptococcus agalactiae* TL2T0256, *Streptococcus anginosus* TL2T0322, *G. vaginalis* TL2D0326, and *Fannyhesseavaginae* (previously known as *Atopobiumvaginae*) TL2B0206 were cultured at 37°C on Columbia blood agar (Thermo Fisher Scientific, Waltham, MA, United States; PB0123A); and *Candida albicans* TL2G0519 and *Candida glabrata* (synonym, *Nakaseomyces glabrata*) TL2G0527 were cultured at 37°C using Sabouraud dextrose broth (Beijing Land Bridge Technology, Beijing, China; CP154A).

#### 2.1.2 Antibacterial tests

Antibacterial tests were performed by following the Clinical and Laboratory Standard Institute guidelines (Cockerill et al., 2012). Briefly, bacterial colonies were transferred to sterile phosphate-buffered saline (PBS). For the drug sensitivity test, the OD_600_ value of the microbial suspension was adjusted to 0.2. To acquire the 5 × 10^5^ CFU/mL inocula, the suspension was diluted 100 times, respectively, and added to a 96-well plate (Biologix, China) with different concentrations of BBA. After that, the plates were cultured at 37°C for 8 h, and bacterial colonies were counted. For the bacteriostatic test, bacterial solutions were added to a liquid medium (as described in Section 2.1.1) with different concentrations of BBA (0, 1, 5, 10, 15, and 20% v/v). The growth curve was determined using a SpectraMax ABS microplate reader (Molecular Devices, Sunnyvale, CA, United States).

Agar medium was poured into a six-well microplate (Corning Inc., Corning, NY, United States; 3516) with 5 mL in each well. One hundred microliters of microbial suspension with an OD_600_ of 0.2 was evenly spread in the six-well plate. After the bacterial suspension was dried, 450 μL of water and gels with different BBA concentrations was separately drawn and evenly spread on the six-well plate. The plate was then incubated at 37°C for 24–48 h. All antibacterial tests were repeated at least three times.

#### 2.1.3 Selection of gel and preparation of BBA gel

We chose ammonium acryloyldimethyltaurate/Vp copolymer (AVC), Carbomer, and Sepimax Zen as the candidate gels. First, they were mixed with BBA to obtain different BBA solutions. The resulting BBA gel solutions were divided into 5-mL clear glass bottles and placed at room temperature for adhesion and fluidity tests. Briefly, 500 μL of BBA gel samples was dropped onto the acrylic plate. The acrylic plate was then tilted 60° horizontally, and the flow state of the gel on the acrylic plate (after 30 min) was observed. Finally, ion tolerance tests were performed to determine the most appropriate gel that showed a proper adhesion effect and tolerance to simulated vaginal salt ions. The simulated vaginal salt ionic solution applied in this study included sodium chloride (2.279 g/L), glycerin (0.49 g/L), albumin (0.009 g/L), potassium chloride (1.752 g/L), sodium acetate (1.805 g/L), and amino acids (0.011 g/L). As shown in Supplementary Figure S1, among these candidate gels, 0.5% AVC (w/v) was the most appropriate gel as it has a good adhesion effect and can be dissolved by simulated vaginal salt ionic solution. BBA gels were prepared by mixing the original solution with 0.5% AVC (w/v) at 37°C under aseptic conditions right before usage.

### 2.2 *In vivo* studies

#### 2.2.1 Mice

Female C57BL/6 mice aged 7–8 weeks were purchased from Jinan Pengyue Company (Jinan, China) and were maintained on a light: dark cycle of 12 h:12 h. The temperature was controlled at approximately 23°C. The mice were provided with free access to food and water. All animal experiments were approved by the Ethics Committee of Tsinghua University (QAU2022040288). All experiments *in vivo* were repeated at least three times.

#### 2.2.2 Intragastric administration

Female mice were randomly divided into three groups: control (0.6 mL of saline), 0.3 mL (0.3 mL of BBA + 0.3 mL of saline), and 0.6 mL (0.6 mL of BBA) groups. The body weights were similar between the three groups. Each group had 30 mice. The volume of 0.6 mL saline or BBA was given to mice by gavage once a day for 4 weeks before sacrifice. Body weight was measured weekly. After intragastric administration, the ovaries (*n* ≥ 7), livers (*n* ≥ 10), spleens (*n* ≥ 10), and kidneys (*n* ≥ 10) were collected and weighed, and the organ index values were calculated as the organ weight/body weight. Oocytes were collected from the ampullae (Chen et al., 2023).

#### 2.2.3 Establishment of vaginitis models

Vaginitis mouse models were generated as previously described (González et al., 2009; Yano and Fidel, 2011; Gilbert et al., 2019; Li et al., 2023). Briefly, female C57BL/6 mice at the age of 8 weeks were randomly divided into two groups: control and vaginitis groups. Each group had 60 mice. For the vaginitis model, mice received a subcutaneous injection of 0.5 mg of 17β-estradiol (E8875-1G; Sigma, St Louis, MO, United States; diluted in corn oil) every 3 days throughout the whole experimental period; 3 days after the first injection, 20 μL of microbial suspension (approximately 5.0 × 10^8^ colony-forming units [CFU]/mL) was intravaginally inoculated.

#### 2.2.4 BBA treatment

When the vaginitis models were successfully established, the mice were further divided into three groups: untreated, treated with PBS or gel (PBS or gel), and treated with BBA or 70% BBA gel for 7 days (BBA or BBA gel). The control group was treated with PBS. After treatment, vagina samples were collected and separated into two parts, with one half used for enzyme-linked immunosorbent assays (ELISAs) and the other half used for hematoxylin and eosin (HE) staining.

We further set three control groups including untreated control (UC), corn oil injection (oil), and 17β-estradiol injection (E) groups, without intravaginal inoculation of microbial suspension.

#### 2.2.5 Immunofluorescence analysis

Following the method by Li et al. (2023), oocytes were fixed with 4% (w/v) paraformaldehyde (PFA) and permeabilized with 0.5% (v/v) Triton X-100 for 20 min. Thereafter, the oocytes were blocked using 1% (w/v) bovine serum albumin for 1 h and incubated with an anti-tubulin antibody (Sigma, T6199) overnight at 4°C. The oocytes were then washed three times and incubated with the secondary antibody at room temperature for 1 h. Fluorescence was examined using a confocal microscope (SP5; Leica, Wetzlar, Germany).

#### 2.2.6 Elisa

The IL-1β, IL-6, and IL-17 concentrations in vaginal tissue were examined using ELISA kits (Jinma, Shanghai, China) according to the manufacturer’s instructions (Yu et al., 2018). Briefly, vaginal tissues were homogenized using an Absolute-Fine Grinder (Monad, Wuhan, China) and a Non-Contact Ultrasonic Crusher (Diagenode SA, Belgium). The kit included all the reagents and plates. After centrifugation, the supernatant was used for ELISAs. A positive control was used to generate the standard curve. Each sample was analyzed in triplicate; 50 μL of the standard, samples, and blank were added to a 96-well plate (Corning, 3596), covered with a sealer, and incubated at room temperature for 2.5 h. The working solution with the antibody was then added and incubated for 1 h. Thereafter, the plates were treated with horseradish peroxidase–streptavidin solution (Thermo Fisher, N100), 3,3′,5,5′-tetramethylbenzidine substrate (Thermo Fisher, N301), and a stop solution. The OD values were measured using an mCD-1oplate reader (BioTek, Winooski, VT, United States) at 450 nm. A four-parameter logistic standard curve was generated to calculate the concentrations. For each group, 20 samples were examined.

#### 2.2.7 HE staining

Tissue damage was evaluated as previously described (Wang et al., 2022). Briefly, vaginal tissues were fixed overnight with 4% paraformaldehyde (PFA). Thereafter, the samples were dehydrated with 50, 70, 90, 95, and 100% ethanol, treated with dimethylbenzene, and embedded in paraffin. Embedded samples were sectioned at a thickness of 5 μm and then distributed on glass slides. The sections were dewaxed and dehydrated, stained with HE, and examined under a microscope (BX51; Olympus, Tokyo, Japan).

### 2.3 Statistical analysis

The data are presented as the mean ± standard deviation, and the statistical significance was examined using a two-tailed Student’s *t*-test, using GraphPad Prism 9.0 (GraphPad, San Diego, CA, United States). The statistical significance of the percentage data was examined using the chi-square test. Differences were considered significant if the *p*-value was <0.05.

## 3 Results

### 3.1 BBA effectively suppressed the proliferation of microorganisms

*L. crispatus* TL1J0136, *L. jensenii* TL1J0062, *L. jensenii* TL1J0081, and *L. iners* TL1J0546 are the dominant species in healthy vaginas. *E. coli* ATCC 25922, *S. aureus* ATCC 25923, *E. faecalis* TL2I0352, *S. agalactiae* TL2T0256, and *S. anginosus* TL2T0322 are common pathogens in AV. *G. vaginalis* TL2D0326 and *A. vaginae* (synonym, *Fannyhesseavaginae*) TL2B0206 are common species in BV. *C. albican*sTL2G0519 and *C. glabrata* (synonym, *Nakaseomyces glabrata*) TL2G0527 are the dominant pathogens causing VVC. As shown in Table 1, the BBA killed all microorganisms exposed for 8 h, and similar results were obtained after diluting the BBA two-fold. However, at dilutions greater than two-fold, the microorganisms could not be completely killed.

**Table 1:** Determination of the bactericidal activity of BBA with different dilutions in contact with all studied microbial strains for 8 h.

BBA concentrations with determination of the bactericidal activity								
Strain	Count (CFU)	BBA with different dilutions						
		0	2	2 × 10^1^	2 × 10^2^	2 × 10^3^	2 × 10^4^	2 × 10^5^
*E. coli ATCC 25922*	8 × 10^5^	−	−	+	+	+	+	+
*S. aureus ATCC 25923*	2 × 10^5^	−	−	+	+	+	+	+
*E. faecalis TL2I0352*	4 × 10^5^	−	−	+	+	+	+	+
*S. agalactiae TL2T0256*	7 × 10^5^	−	−	+	+	+	+	+
*S. anginosus TL2T0322*	4 × 10^5^	−	−	+	+	+	+	+
*G. vaginalis* TL2D0326	8 × 10^4^	−	−	+	+	+	+	+
*A. vaginae TL2B0206*	3 × 10^5^	−	−	+	+	+	+	+
*C. albicans TL2G0519*	1 × 10^5^	−	−	+	+	+	+	+
*C. glabrata TL2G0527*	2 × 10^5^	−	−	+	+	+	+	+
*L. crispatus TL1J0136*	6 × 10^5^	−	−	+	+	+	+	+
*L. jensenii TL1J0062*	2 × 10^5^	−	−	+	+	+	+	+
*L. gasseri TL1J0081*	9 × 10^5^	−	−	+	+	+	+	+
*L. iners TL1J0546*	2 × 10^5^	−	−	+	+	+	+	+

“+” indicates the microorganisms were not completely killed; “−” indicates the microorganisms were 100% killed.

**Figure 1:**
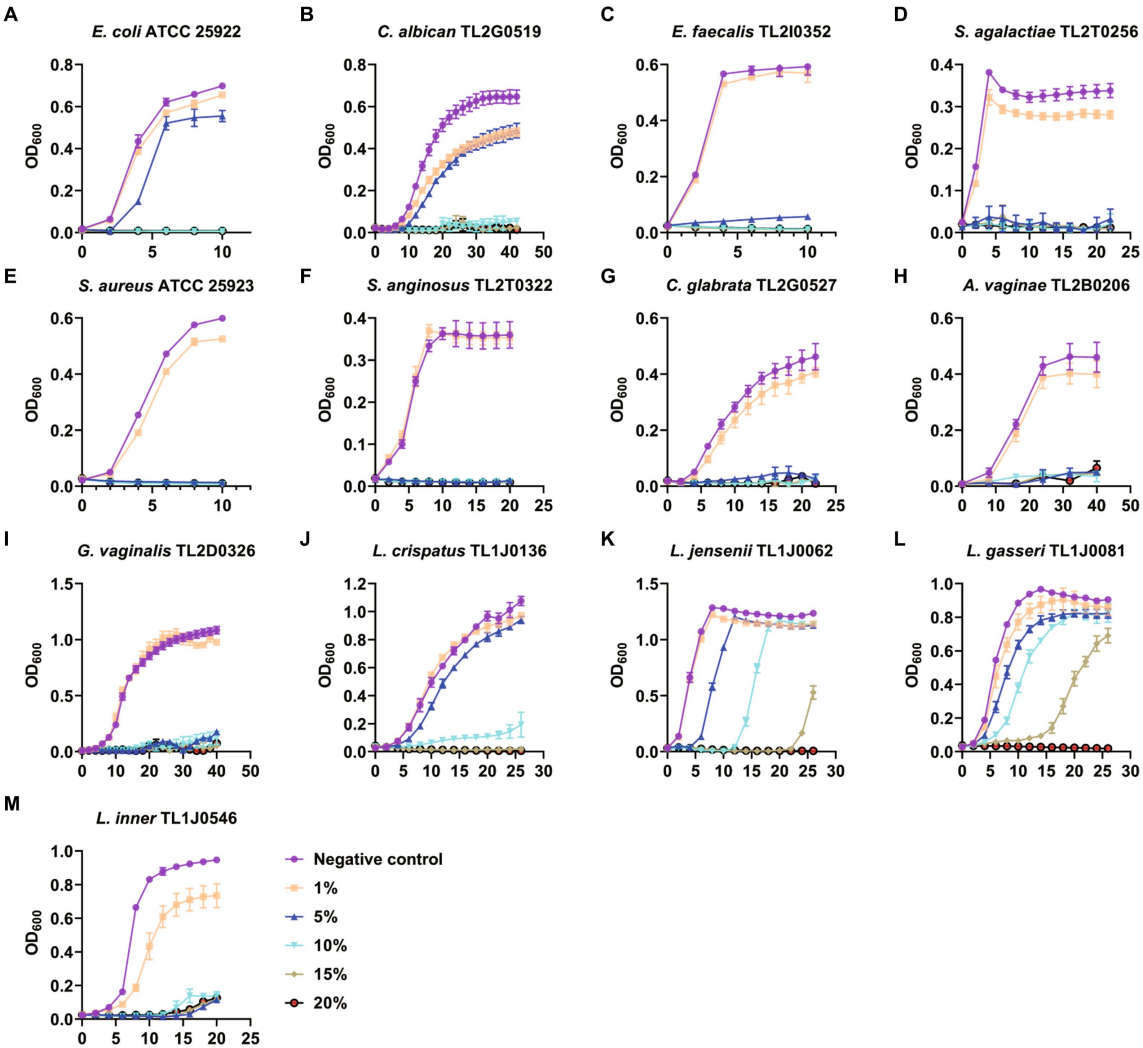
Antibacterial effect of BBA. BBA, biological bacteriostatic agent. **(A)** *Escherichia coli*. **(B)** *Candida albican*. **(C)** *Enterococcus faecalis*. **(D)** *Streptococcus agalactiae*. **(E)** *Staphylococcus aureus*. **(F)** *Streptococcus anginosus*. **(G)** *Candida glabrata*. **(H)** *Atopobium vaginae*. **(I)** *Gardnerella vaginalis*. **(J)** *Lactobacillus crispatus*. **(K)** *Lactobacillus jensenii*. **(L)** *Lactobacillus gasseri*. **(M)** *Lactobacillus inner*.

Therefore, we examined the effect of BBA on microbial growth. Media with different concentrations of BBA were used to culture the microorganisms. The growth of *E. coli* ATCC 25922 and *C. albicans* TL2G0519 was almost completely suppressed when the BBA concentration was >10% (Figures 1A,B). The growth of other pathogens was effectively suppressed by 5% BBA (Figures 1C–I). The growth of probiotics, *L. crispatus* TL1J0136, *L. jensenii* TL1J0062, and *L. gasseri* TL1J0081 was not affected at BBA concentrations lower than 10% (Figures 1J–L), but the growth of *L. iners* TL1J0546 was suppressed at a concentration of 5% (Figure 1M). These results suggested that BBA at a concentration of 5% can effectively inhibit most pathogens, but most probiotics were not significantly affected.

### 3.2 BBA had no deleterious effects on organ indices

**Figure 2:**
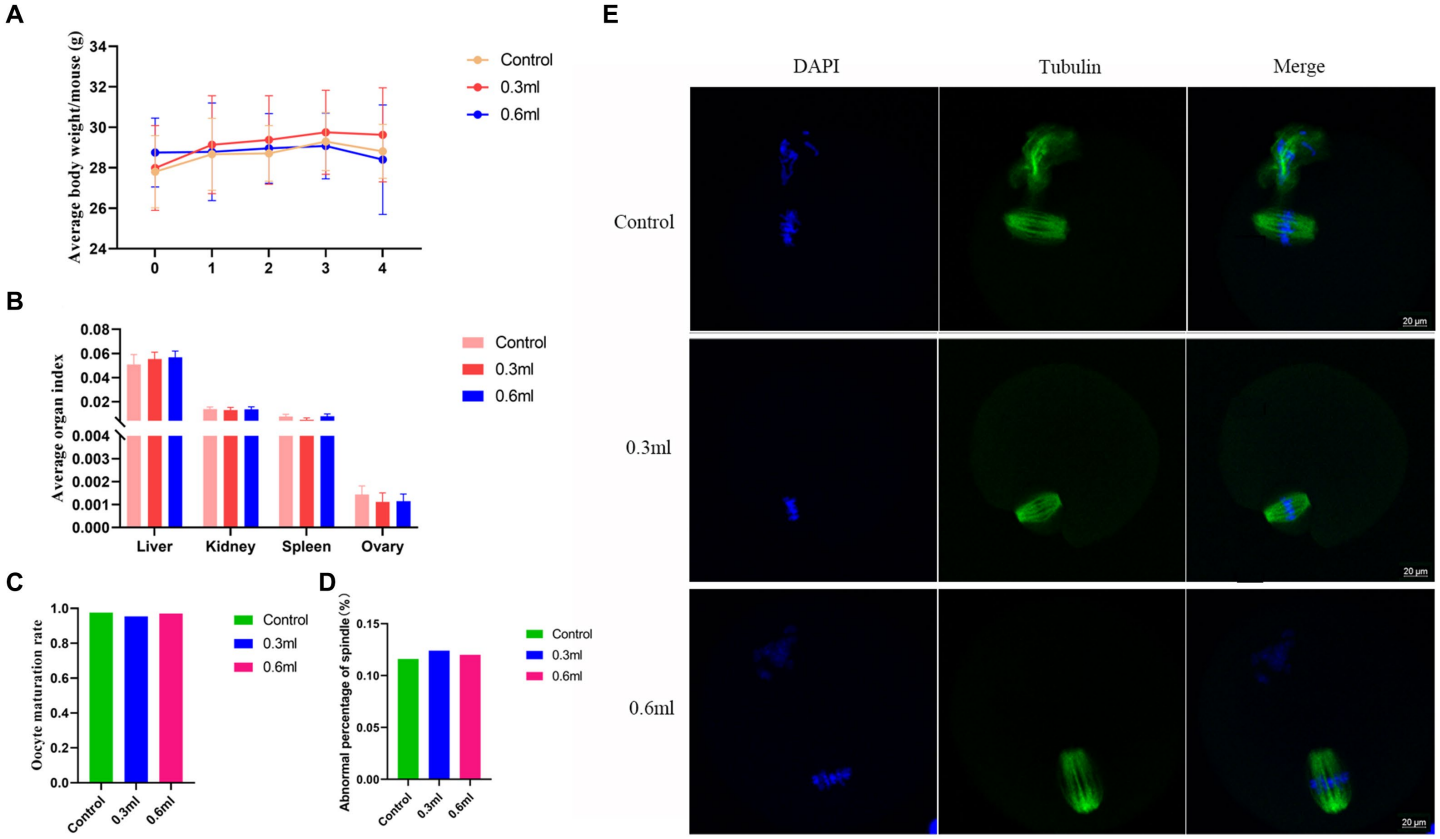
Effects of BBA on organ index values. **(A)** Average body weight. **(B)** Organ index values after intragastric administration of BBA. **(C–E)** Oocyte maturation and spindle assembly were not affected by BBA. Bar, 20 μm. The error bars are standard deviations. BBA, biological bacteriostatic agent.

To investigate whether BBA has adverse effects on the organs, female C57BL/6 mice (7–8 weeks) were intragastrically administered BBA at different doses for 4 weeks. The average body weight was not significantly affected by BBA compared to the body weight of the control mice (Figure 2A). The organ index values of the liver, spleen, kidneys, and ovaries were similar among the groups (Figure 2B). We further examined the effects of BBA on oocyte maturation and found that oocyte maturation and spindle organization were not significantly influenced by BBA (Figures 2C–E). These results indicated that BBA is safe.

### 3.3 BBA effectively alleviated inflammation in mice with *Candida albicans*-induced vaginitis

**Figure 3:**
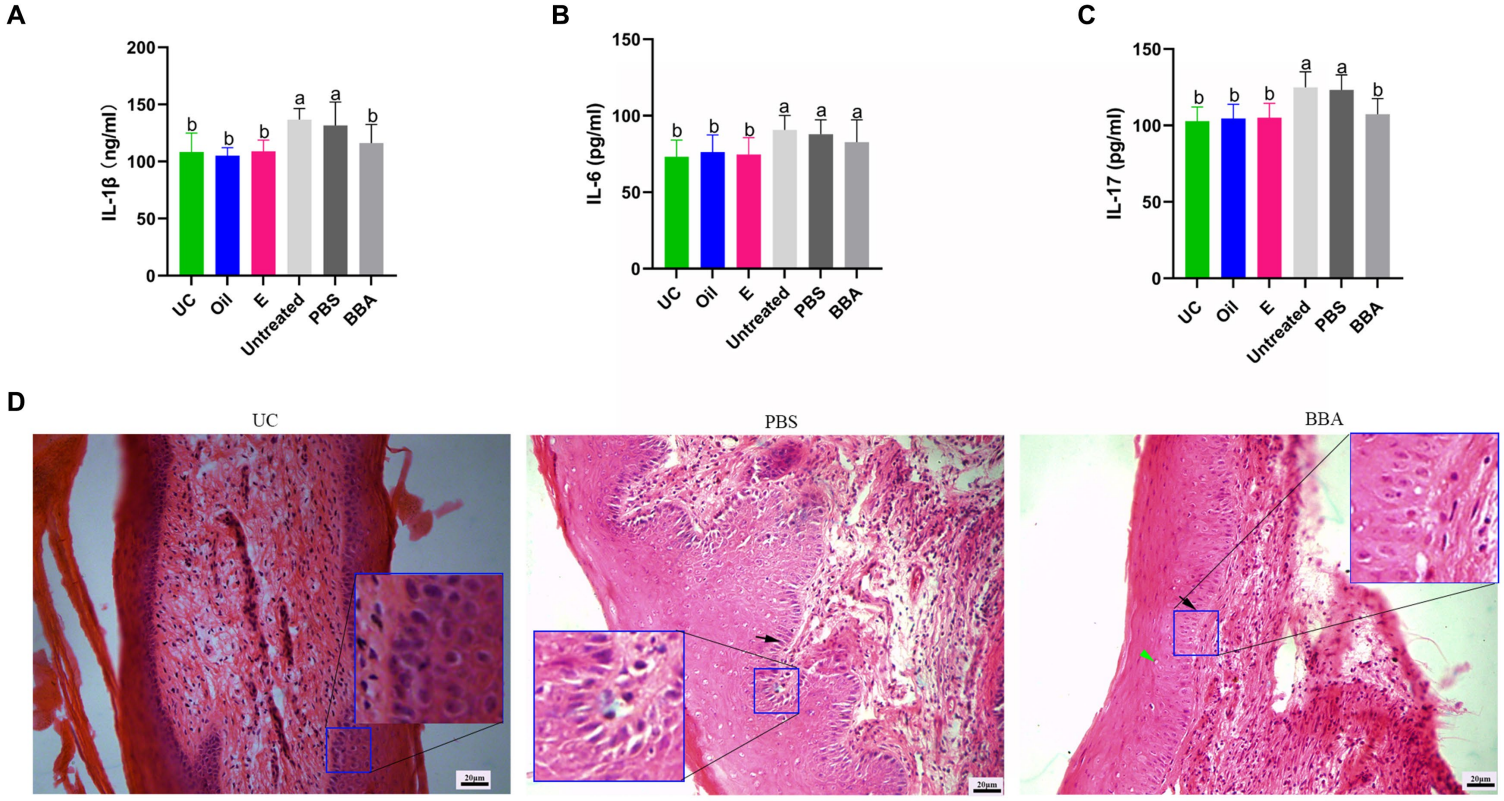
Efficacy of BBA against *C. albicans*-induced vaginitis in mice. **(A–C)** Levels of IL-1β, IL-6, and IL-17 in vaginal tissues. **(D)** HE staining of vaginal tissues. Green arrow, cavitation; black arrow, epithelial cells. The same letter indicates no significant difference between groups; different letters indicate a significant difference between groups. The error bars are standard deviations. BBA, biological bacteriostatic agent; IL-1β, interleukin-1β; IL-6, interleukin-6; IL-17, interleukin-17; HE staining, hematoxylin and eosin staining.

To confirm the efficacy of BBA against vaginitis, we used a *C. albicans*-induced vaginitis model using C57BL/6 mice as described previously (Ma et al., 2019; Zhao et al., 2022). After treatment with BBA for 7 days, we examined the vaginal fungal burden (CFU) in vaginal lavage samples and found no *C. albicans* in mice in the uninfected control group. The fungal burdens were approximately (8.36 ± 4.007) × 10^5^ CFU/mL and (7.73 ± 5.216) × 10^5^ CFU/mL in the PBS and BBA groups, respectively. *C. albicans*-induced vaginitis significantly increased the levels of interleukin (IL) 1β, IL-6, and IL-17 in vaginal tissues, but the levels of IL-1β and IL-17 were significantly reduced by BBA treatment (Figures 3A–C). *C. albicans* impaired the vaginal mucosa, which was improved by BBA treatment (Figure 3D). These results suggested that BBA effectively reduced inflammation in mice with *C. albicans*-induced vaginitis.

### 3.4 BBA effectively alleviated inflammation in mice with *Candida glabrata*-induced vaginitis

**Figure 4:**
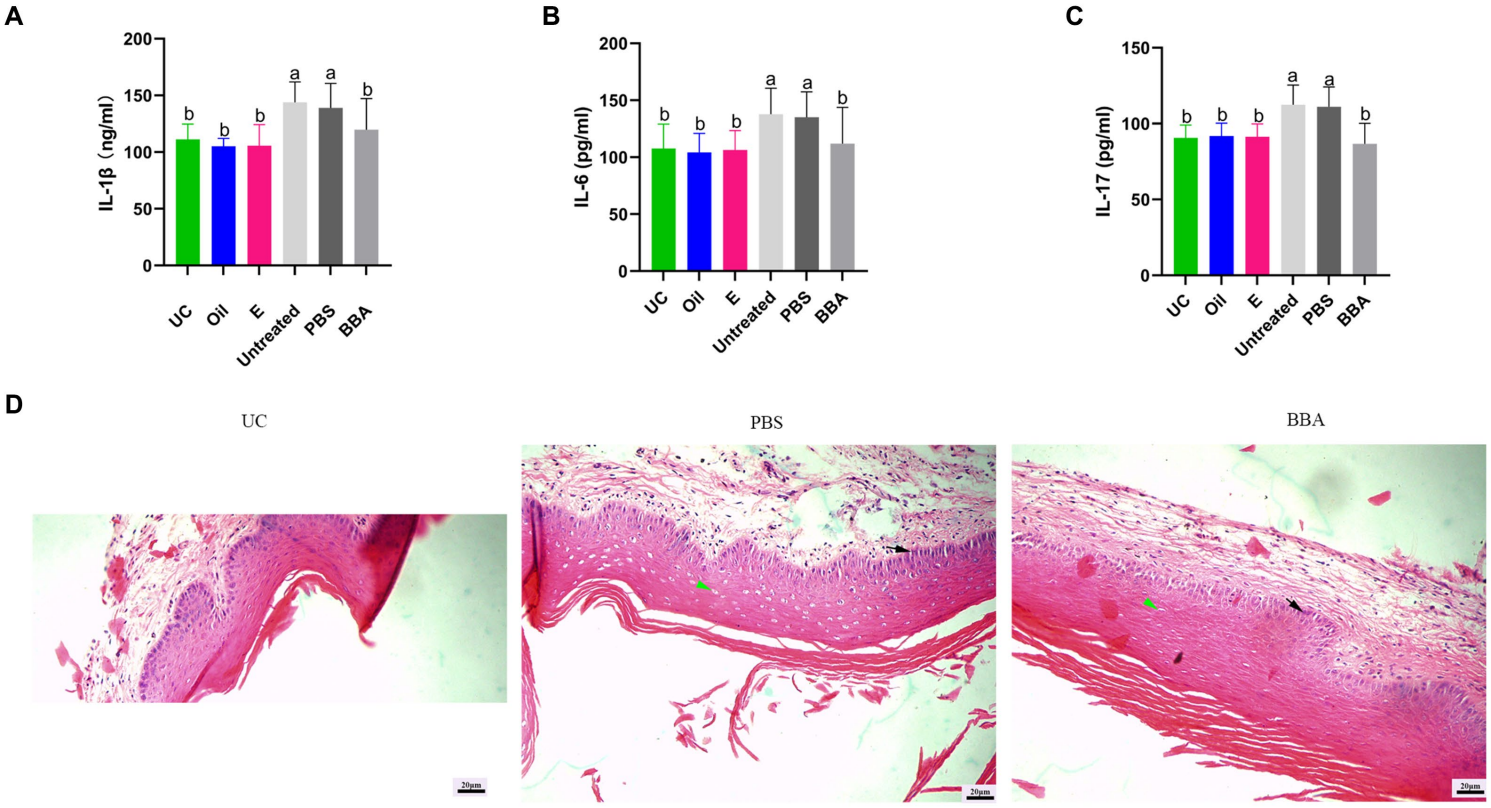
Efficacy of BBA against *C. glabrata*-induced vaginitis. **(A–C)** Concentrations of IL-1β, IL-6, and IL-17 in vaginal tissues. **(D)** HE staining of vaginal tissues. Green arrow, cavitation; black arrow, epithelial cells. The same letter indicates no significant difference between groups; different letters indicate a significant difference between groups. The error bars are standard deviations. BBA, biological bacteriostatic agent; IL-1β, interleukin-1β; IL-6, interleukin-6; IL-17, interleukin-17; HE, hematoxylin and eosin.

*C. glabrata*-induced vaginitis is resistant to antifungal agents. We investigated the efficacy of BBA against *C. glabrata*-induced vaginitis in a mouse model. After treatment with BBA, the bacteria densities reduced to approximately (4 ± 2.872) × 10^5^ CFU/mL from approximately (7.33 ± 4.097) × 10^5^ CFU/mL (*p* = 0.0512) in vaginal lavage samples. The increased levels of IL-1β, IL-6, and IL-17 were also reduced by BBA treatment (Figures 4A–C). In addition, we found that the vaginal mucosa was impaired. After treatment with BBA, the impaired vaginal mucosa was partially repaired and inflammation was reduced. The number and size of cavitations were also decreased by BBA treatment (Figure 4D). These results suggested that BBA alleviates inflammation in *C. glabrata*-induced vaginitis.

### 3.5 BBA partly improved inflammation in a mouse model of *Gardnerella vaginalis*-induced vaginitis

**Figure 5:**
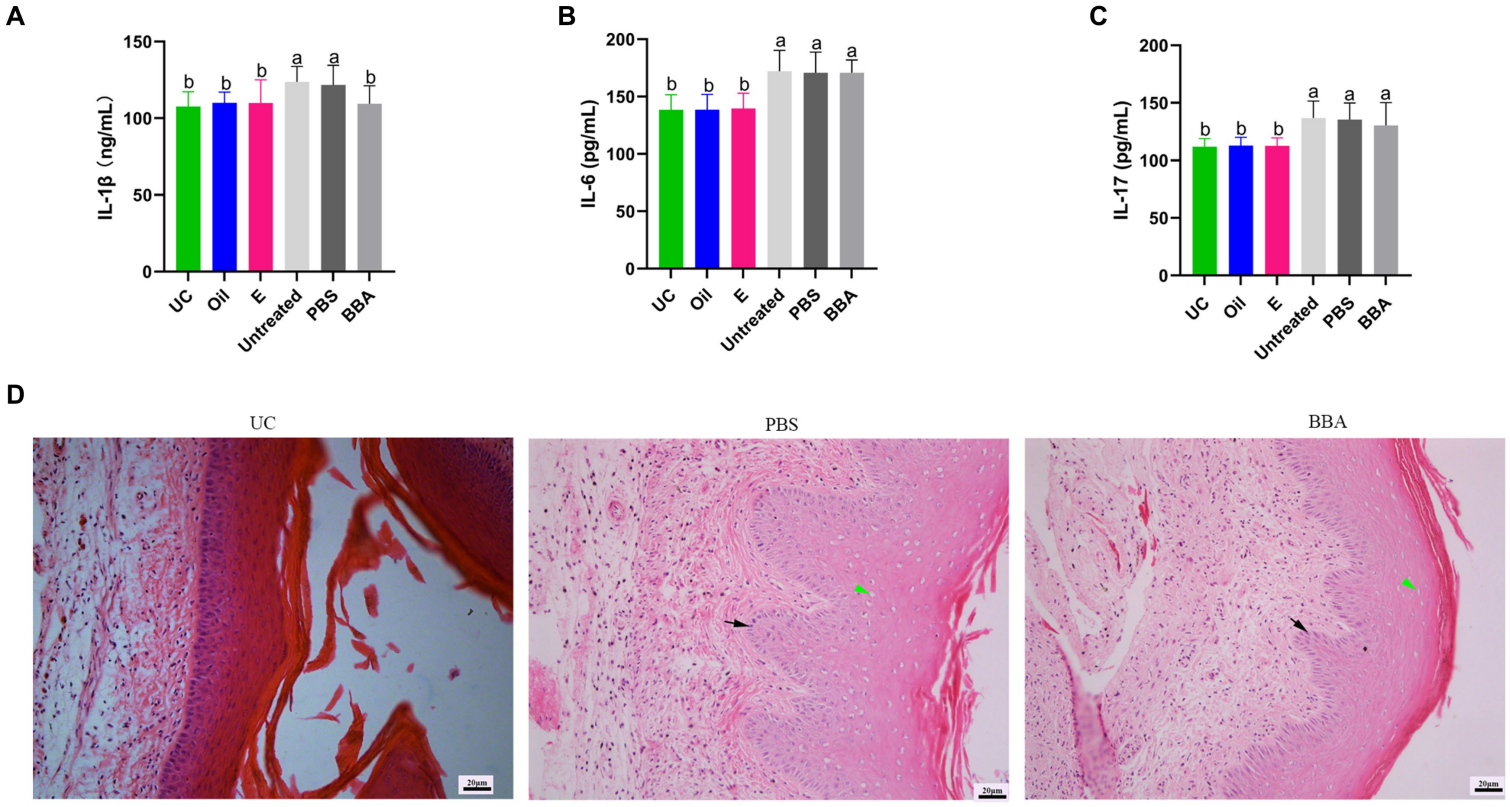
BBA partly improved *Gardnerella vaginalis-induced inflammation*. **(A–C)** Concentrations of IL-1β, IL-6, and IL-17 in vaginal tissues. **(D)** HE staining of vaginal tissues. Green arrow, cavitation; black arrow, epithelial cells. The same letter indicates no significant difference between groups; different letters indicate a significant difference between groups. The error bars are standard deviations. BBA, biological bacteriostatic agent; IL-1β, interleukin-1β; IL-6, interleukin-6; IL-17, interleukin-17; HE, hematoxylin and eosin.

BV is one of the most common types of vaginitis in women of reproductive age. *G. vaginalis* is one of the most common microorganisms associated with BV infections. We used a mouse model to investigate the efficacy of BBA against vaginitis induced by *G. vaginalis*. We found that BBA significantly reduced the level of IL-1β in vaginal tissues, but the concentrations of IL-6 and IL-17 were similar between groups (Figures 5A–C). The HE staining results showed that the impaired vaginal mucosa was partly improved after BBA treatment (Figure 5D). These results indicated that BBA has therapeutic effects against vaginitis induced by *G. vaginalis*.

### 3.6 The BBA gel effectively suppressed the growth of microorganisms

The BBA is a liquid formulation that is prone to leakage from the vagina. Therefore, we prepared a BBA gel and investigated its efficacy against common vaginal microbes. As described in the Methods section, gels were prepared by combining various excipients (Sepimax Zen, AVC, and Carbomer) and BBA in different proportions, and the fluidity, viscosity, salt ion resistance, and antibacterial activity of the gels were evaluated. We confirmed that 0.5% AVC can be used as an excipient to prepare BBA gels. To investigate the effect of the excipients on the antibacterial activity of BBA, we evaluated the antibacterial activity of gels with different BBA contents. As shown in Table 2, the gel with 50% BBA suppressed the growth of most microorganisms, except *E. faecalis TL2I0352*, *S. agalactiae TL2T0256*, *C. albicans TL2G0519*, and *C. glabrata TL2G0527*. However, the growth of all microorganisms was suppressed by the gel containing 70% BBA. These results indicated that the gel with 70% BBA had a satisfactory bacteriostatic effect. Therefore, gels containing 70% BBA were used for further experiments (Supplementary Figure S2).

**Table 2:** Inhibitory effect of gels with different contents of BBA on the growth of different strains.

Strain	Bacteria count (CFU)	Content of BBA in gel (%)				
		0	10	30	50	70
*E. coli* ATCC 25922	×10^6^	+	+	+	−	−
*S. aureus* ATCC 25923	×10^6^	+	+	+	−	−
*E. faecalis* TL2I0352	×10^6^	+	+	+	+	−
*S. agalactiae* TL2T0256	×10^6^	+	+	+	+	−
*S. anginosus* TL2T0322	×10^6^	+	+	+	−	−
*G. vaginalis* TL2D0326	×10^6^	+	+	+	−	−
*A. vaginae* TL2B0206	×10^6^	+	+	+	−	−
*C. albicans* TL2G0519	×10^6^	+	+	+	+	−
*C. glabrata* TL2G0527	×10^6^	+	+	+	+	−
*L. crispatus* TL1J0136	×10^6^	+	+	+	−	−
*L. gasseri* TL1J0081	×10^6^	+	+	+	−	−
*L. jensenii* TL1J0062	×10^6^	+	+	+	−	−
*L. iners* TL1J0546	×10^6^	+	+	+	+	−

BBA, biological bacteriostatic agent; *E. coli, Escherichia coli; S. aureus, Staphylococcus aureus; E. faecalis, Enterococcus faecalis; S. agalactiae, Streptococcus agalactiae; S. anginosus, Streptococcus anginosus; G. vaginalis, Gardnerella vaginalis; A. vaginae, Atopobiumvaginae; C. albicans, Candida albicans; C. glabrata, Candida glabrata; L. crispatus, Lactobacillus crispatus; L. jensenii, Lactobacillus jensenii; L. gasseri, Lactobacillus gasseri; L. iners, Lactobacillus iners*; CFU, colony-forming unit.

### 3.7 The 70% BBA gel significantly reduced inflammation associated with VVC

**Figure 6:**
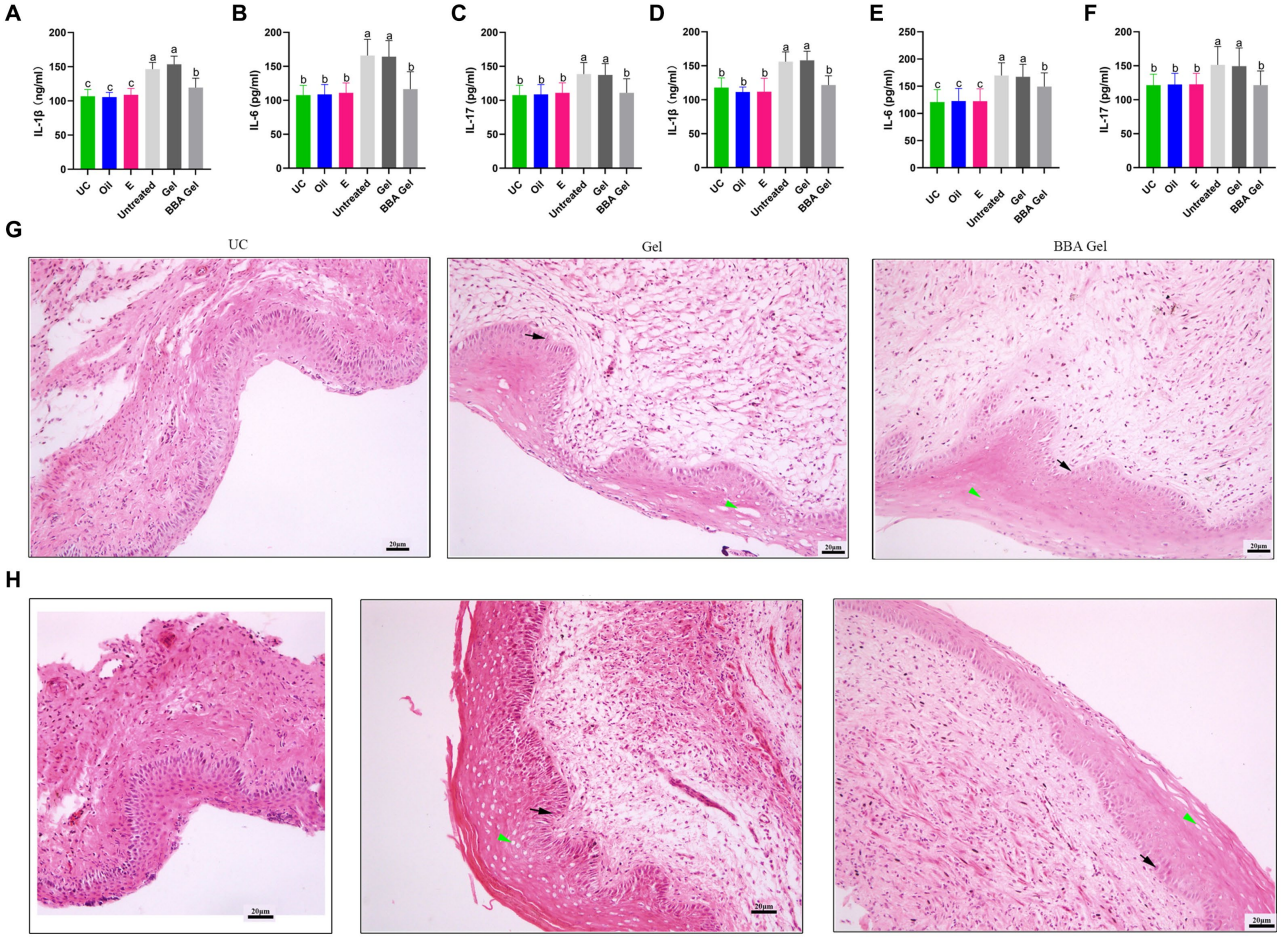
Efficacy of a 70% BBA gel against VVC. **(A–F)** Concentrations of IL-1β, IL-6, and IL-17 in vaginal tissues of mice with *C. albicans*- and *C. glabrata*-induced vaginitis. **(D)** Vaginal tissues were stained to further investigate the role of the 70% BBA gel in the treatment of VVC. Green arrow, cavitation; black arrow, epithelial cells. The same letter indicates no significant difference between groups; different letters indicate a significant difference between groups. The error bars are standard deviations. BBA, biological bacteriostatic agent; VVC, vulvovaginal candidiasis; IL-1β, interleukin-1β; IL-6, interleukin-6; IL-17, interleukin-17; C. albicans, Candida albicans; C. glabrata, Candida glabrata. **(G,H)** HE staining of vaginal tissues from mice with *C. albicans*- and *C. glabrata*-induced vaginitis.

We further constructed mouse models of vaginitis induced by *C. albicans* and *C. glabrata* to investigate the efficacy of the 70% BBA gel. We found that the gel significantly decreased the concentrations of IL-1β, IL-6, and IL-17 in vaginal tissues (Figures 6A–F). The HE staining results also indicated that the impaired vaginal mucosa was improved in both vaginitis models by treatment with the 70% BBA gel (Figures 6G,H). These results suggested that the 70% BBA gel can effectively alleviate inflammation associated with VVC.

### 3.8 *Gardnerella vaginalis*-induced inflammation was improved by the 70% BBA gel

**Figure 7:**
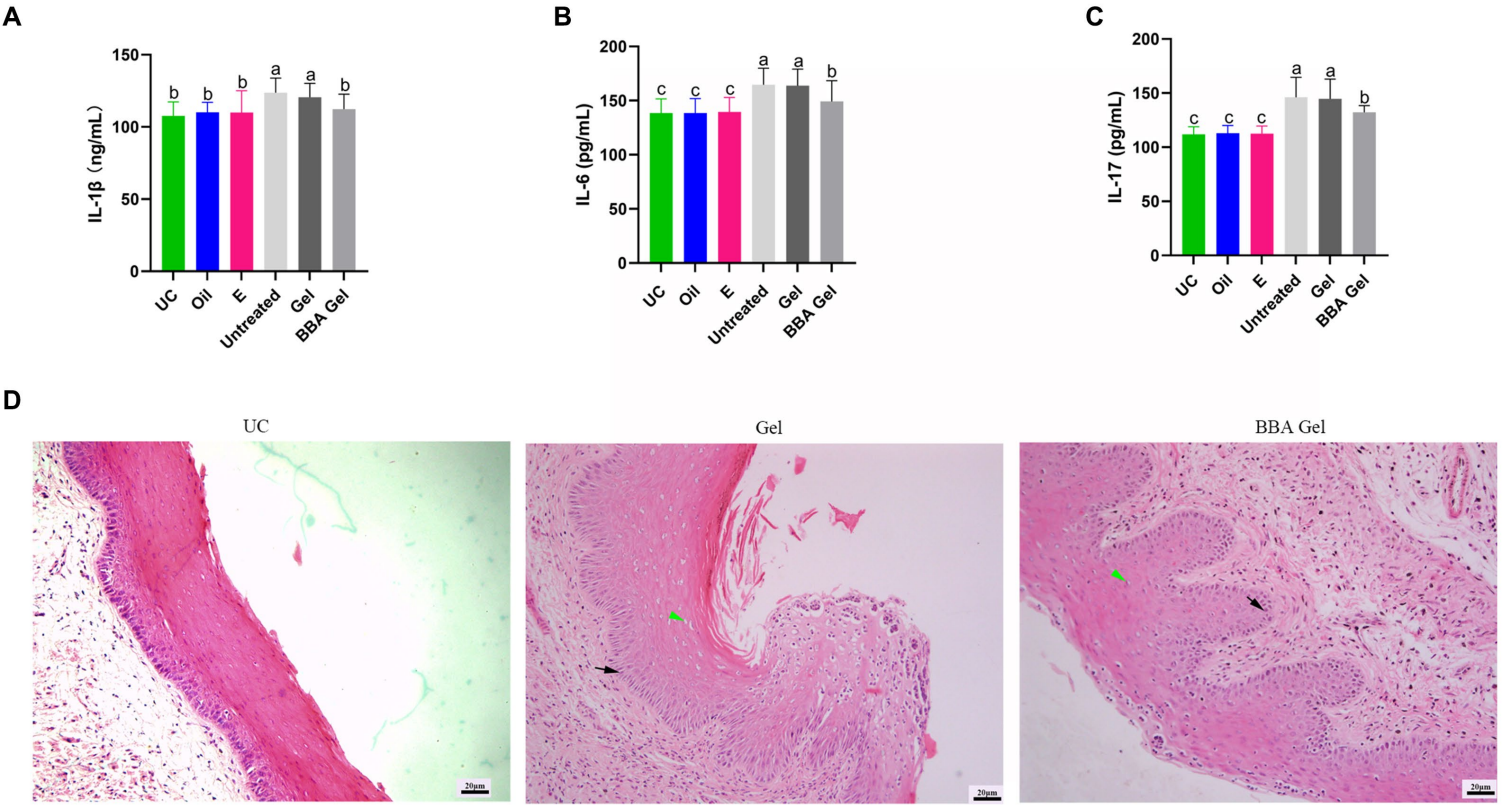
70% BBA gel alleviated *Gardnerella vaginalis-induced inflammation*. **(A–C)** Concentrations of IL-1β, IL-6, and IL-17 in vaginal tissues. **(D)** Representative images showing the effects of the 70% BBA gel on vaginal inflammation induced by *Gardnerella vaginalis*. Green arrow, cavitation; black arrow, epithelial cells. The same letter indicates no significant difference between groups; different letters indicate a significant difference between groups. The error bars are standard deviations. BBA, biological bacteriostatic agent; IL-1β, interleukin-1β; IL-6, interleukin-6; IL-17, interleukin-17.

We also examined the role of 70% BBA gel in the treatment of *G. vaginalis*-induced inflammation and found that the gel significantly decreased the concentrations of IL-1β, IL-6, and IL-17 in vaginal tissues (Figures 7A–C). The HE staining results also demonstrated that the 70% BBA gel was effective at repairing the impaired vaginal mucosa (Figure 7D). These results indicated that the 70% BBA gel effectively improved *G. vaginalis*-induced inflammation.

## 4 Discussion

Vaginal infection is a prevalent medical problem in women, with 75% of women experiencing at least one episode of vaginitis during their lifetime. However, it is challenging to completely cure vaginitis, especially recurrent vaginitis, owing to the resistance of the pathogens to agents and the formation of biofilms. In this study, we investigated the efficacy of BBA formulated from natural products against vaginitis. BBA alone and the 70% BBA gel had significant bacteriostatic effects *in vitro* and effectively improved inflammation in mouse models of VVC and BV.

Compared to the microbiome of the skin, gut, or mouth, the healthy vaginal microbiome is also a complex and dynamic ecosystem containing 70–90% lactobacilli, *G. vaginalis*, *E. coli*, group *B Streptococcus*, genital *Mycoplasma* species, and *C. albicans* (Mitchell et al., 2015; Smith and Ravel, 2017; Marnach et al., 2022). BV is associated with a shift in the vaginal microbiome toward the overgrowth of *G. vaginalis* and *Atopobiumvaginae*. VVC is mainly caused by *C. albicans* (80–92%), *C. glabrata*, *C. parapsilosis*, and other strains. AV is often caused by *Escherichia coli, Enterococcus* spp.*, Streptococcus angina,* and *Streptococcus agalactiae*. The presence of various pathogens causing different kinds of vaginitis poses challenges in selecting specific antimicrobial agents, especially considering the confusing clinical manifestations and unsatisfactory laboratory tests.

Metronidazole, clindamycin, and azoles are commonly used to treat vaginitis (Yadav et al., 2019; Marnach et al., 2022). However, it is difficult to select the specific agents based on clinical manifestations and there are problems with drug resistance. In the present study, we investigated the efficacy of BBA (formulated using natural products) and a BBA gel in inhibiting the growth of *L. crispatus* TL1J0136, *L. jensenii* TL1J0062, *L. gasseri* TL1J0081, *L. iners* TL1J0546, *E. coli* ATCC 25922, *S. aureus* ATCC 25923, *E. faecalis* TL2I0352, *S. agalactiae* TL2T0256, *S. anginosus* TL2T0322, *G. vaginalis* TL2D0326, *G. vaginae* TL2B0206, *C. albicans* TL2G0519, and *C. glabrata* TL2G0527 and found that the growth of these microorganisms was effectively suppressed by the BBA and a 70% BBA gel *in vitro*. BBA has demonstrated effectiveness against a broad range of microbial pathogens, suggesting its potential utility for clinical vaginitis cases involving unclear or multiple pathogens in the future.

Vaginal drug delivery has long been used to treat vaginal infections (das Neves and Bahia, 2006). A wide variety of pharmaceutical forms exist, including tablets, capsules, liquid preparations, and vaginal films (Vermani and Garg, 2000). Several studies have demonstrated that gels are ideal vaginal drug delivery systems (das Neves and Bahia, 2006). In the present study, we found that a liquid BBA preparation and a BBA gel alleviated the inflammation associated with vaginitis in mouse models, but the 70% BBA gel had a greater effect.

In summary, BBA effectively inhibited the growth of microorganisms *in vitro* and improved vaginal inflammation in a mouse model; thus, the development of clinical treatment agents for vaginitis could be expected in the future. However, there are some limitations to this study. First, the mechanisms by which BBA reduces inflammation associated with vaginitis were not investigated. In addition, the effective component of BBA remains to be elucidated and patient-based studies are required to confirm its clinical efficacy against vaginitis.

## Data availability statement

The original contributions presented in the study are included in the article/Supplementary material, further inquiries can be directed to the corresponding authors.

## Ethics statement

The animal study was approved by The Ethics Committee of Tsinghua University (THU-02-2023-0202A). The study was conducted in accordance with the local legislation and institutional requirements.

## Author contributions

ZZ: Data curation, Formal analysis, Investigation, Methodology, Visualization, Writing – original draft, Writing – review & editing. PL: Formal analysis, Investigation, Methodology, Writing – original draft. JL: Data curation, Formal analysis, Software, Writing – original draft. XL: Software, Validation, Visualization, Writing – original draft. ML: Investigation, Methodology, Writing – review & editing. YW: Investigation, Visualization, Writing – review & editing. MZ: Data curation, Visualization, Writing – review & editing. YC: Data curation, Validation, Writing – review & editing. QL: Data curation, Validation, Writing – review & editing. ZG: Methodology, Writing – review & editing. LZ: Conceptualization, Funding acquisition, Resources, Supervision, Writing – review & editing.

## Supplementary material

FIGURE S1 Selection of gel. **(A)** Candidate gels. **(B)** Adhesion tests of candidate gels in **(A)**. **(C)** Tolerance tests of AVC.

FIGURE S2 Anti-bacterial activity of BBA gel. **(A)** *Escherichia coli* ATCC 25922. **(B)** *Staphylococcus aureus* ATCC 25923. **(C)** *Enterococcus faecalis* TL2I0352. **(D)** *Streptococcus agalactiae* TL2T0256. **(E)** *Streptococcus anginosus* TL2T0322. **(F)** *Gardnerella vaginalis* TL2D0326. **(G)** *Atopobium vaginae* TL2B0206. **(H)** *Candida albicans*TL2G0519. **(I)** *Candida glabrata* TL2G0527. **(J)** *Lactobacillus crispatus* TL1J0136. **(K)** *Lactobacillus gasseri* TL1J0081. **(L)** *Lactobacillus jensenii* TL1J0062. **(M)** *Lactobacillus iners* TL1J0546.
