# Supplementary figures and images for: A non-antibiotic antimicrobial drug, a biological bacteriostatic agent, is useful for treating aerobic vaginitis, bacterial vaginosis, and vulvovaginal candidiasis
Source: Front Microbiol. 2024 May 27;15:1341878. doi: 10.3389/fmicb.2024.1341878 (PMC11163058; doi:10.3389/fmicb.2024.1341878)

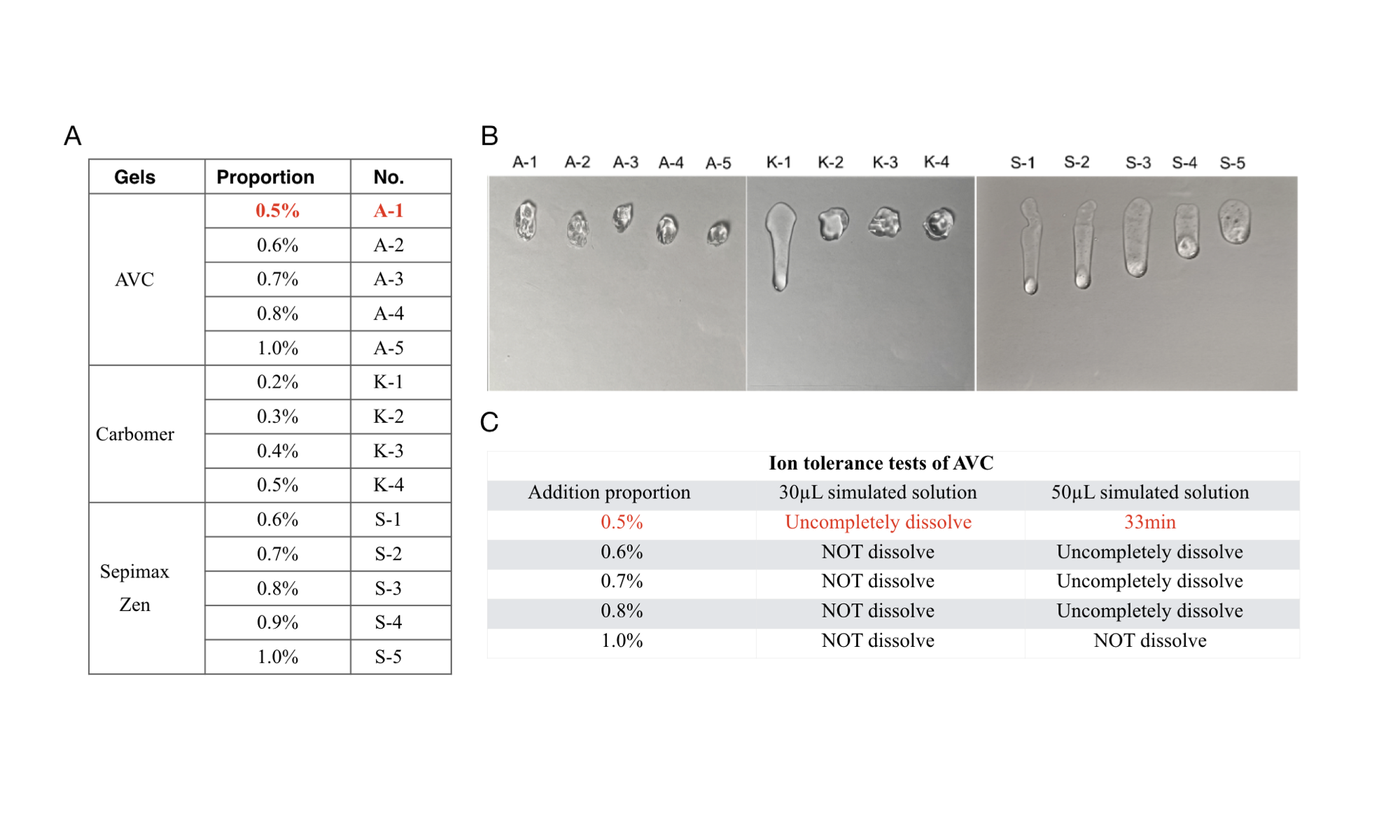

Supplement: FIGURE S1 — Selection of gel. (A) Candidate gels. (B) Adhesion tests of candidate gels in (A). (C) Tolerance tests of AVC. [file Image_1.TIFF]

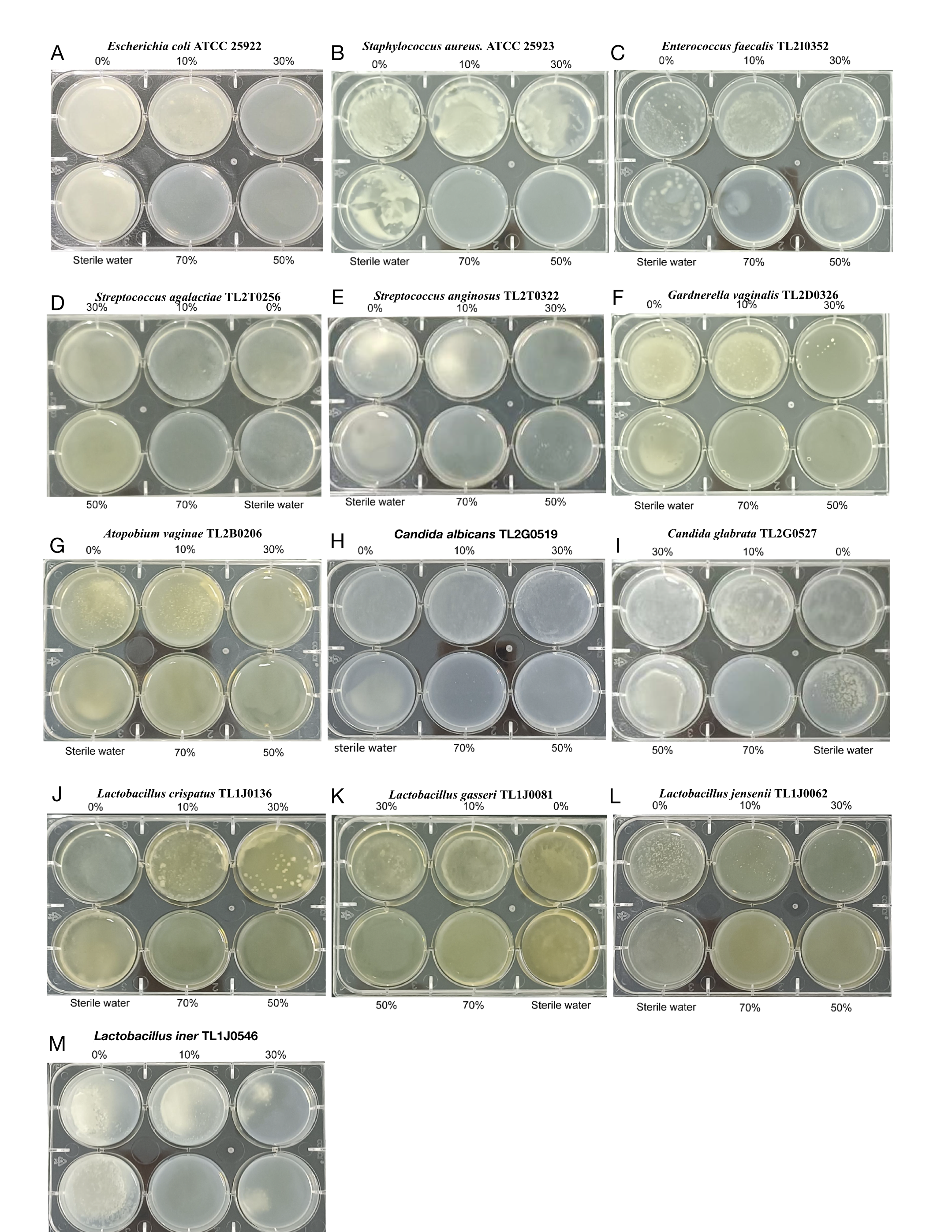

Supplement: FIGURE S2 — Anti-bacterial activity of BBA gel. (A) Escherichia coli ATCC 25922. (B) Staphylococcus aureus ATCC 25923. (C) Enterococcus faecalis TL2I0352. (D) Streptococcus agalactiae TL2T0256. (E) Streptococcus anginosus TL2T0322. (F) Gardnerella vaginalis TL2D0326. (G) Atopobium vaginae TL2B0206. (H) Candida albicansTL2G0519. (I) Candida glabrata TL2G0527. (J) Lactobacillus crispatus TL1J0136. (K) Lactobacillus gasseri TL1J0081. (L) Lactobacillus jensenii TL1J0062. (M) Lactobacillus iners TL1J0546. [file Image_2.TIFF]
